# Switchable Adhesion: On‐Demand Bonding and Debonding

**DOI:** 10.1002/advs.202200264

**Published:** 2022-03-01

**Authors:** Ziyang Liu, Feng Yan

**Affiliations:** ^1^ Jiangsu Engineering Laboratory of Novel Functional Polymeric Materials College of Chemistry Chemical Engineering and Materials Science Soochow University Suzhou 215123 China

**Keywords:** contact mechanics, programmable bonding/debonding, smart materials, soft robotics, switchable adhesion

## Abstract

Adhesives have a long and illustrious history throughout human history. The development of synthetic polymers has highly improved adhesions in terms of their strength and environmental tolerance. As soft robotics, flexible electronics, and intelligent gadgets become more prevalent, adhesives with changeable adhesion capabilities will become more necessary. These adhesives should be programmable and switchable, with the ability to respond to light, electromagnetic fields, thermal, and other stimuli. These requirements necessitate novel concepts in adhesion engineering and material science. Considerable studies have been carried out to develop a wide range of adhesives. This review focuses on stimuli‐responsive material‐based adhesives, outlining current research on switchable and controlled adhesives, including design and manufacturing techniques. Finally, the potential for smart adhesives in applications, and the development of future adhesive forms are critically suggested.

## Graphical abstract

In this review, the authors focused on stimuli‐responsive materials‐based adhesives, summarizing the current works of switchable and controllable adhesives, including the design and fabrication strategies. Finally, the challenges and opportunities for smart adhesives in applications and future forms of adhesives are discussed.

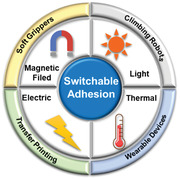

## 1 Introduction

**Figure 1:**
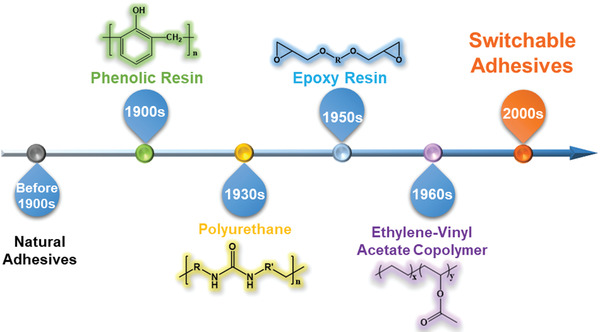
Chronology of significant advancements in adhesives.

Bonding is a practice that dates all the way back to the dawn of humankind. Nature bonding phenomena prompted human to utilize the bonding method. Humans have used water and clay to connect stones and wood for tools as far back as 5000 years ago, but their low adhesive strength and poor environmental tolerance limited their uses.^[^ ^1^ ^]^ Phenolic resins were not created until the early twentieth century to replace natural adhesives.^[^ ^2^ ^]^ Thus far, adhesive development has entered a new phase. Until the 1930s, a plethora of novel adhesives with synthetic polymer materials as the primary component developed as a result of the advent of polymer materials, modern manufacturing, and particularly the demands of the developing aviation sector. Adhesives were initially utilized in airplane structural components in 1941.^[^ ^3^ ^]^ Epoxy resin glue has been around since the 1950s. Epoxy resin is a strong, versatile, and adaptable glue.^[^ ^4^ ^]^ Epoxy resin is now the primary structural glue. Eastman Kodak created cyanoacrylate‐based drying glue in 1957, which we use today.^[^ ^5^ ^]^ Environmental concerns made hot melt glue one of the fastest‐growing adhesives in the 1960s,^[^ ^6^ ^]^ and then followed the second and third generations of acrylic adhesives.^[^ ^7^ ^]^ After 1980s, adhesive studies focused on improving existing adhesives. Modern adhesives have acquired great adhesive strength and other characteristics (such as the tolerance against water, temperature, aging, and so on) (**Figure** **1**).

During the 21st century, adhesives are vital in modern technology and business. For example, in robotics, on‐demand bonding and debonding allow the robot arm to grip and release things.^[^ ^8^, ^9^, ^10^, ^11^ ^]^ Robots based on switchable adhesive can climb and stay on the wall, which attract more and more attention.^[^ ^9^, ^12^ ^]^ In smart printing systems, switchable adhesive can transfer printing small‐scale micro‐nano materials onto arbitrary substrates to fabricate integrated semiconductor devices.^[^ ^13^, ^14^, ^15^, ^16^ ^]^ Moreover, to re‐bond some parts that are not bonded correctly, for example, in the manufacture of electrical devices, they must be detached or pulled apart. Interlacement occurs when the adhesion strength is reduced, or the bonded components are damaged. Electric‐skins with flexibility and sensing ability are designed for attaching to human skin. Without switchable adhesion, the de‐attachment of electric‐skins may cause damage to both humans and devices.^[^ ^17^, ^18^, ^19^, ^20^, ^21^ ^]^ Therefore, modern‐day switchable adhesives are necessary. Switchable adhesives are reversible and controlled, indicating that it may be regulated to bond and de‐bond on demand by external stimuli. Reversible adhesives can be reused several times rather than becoming useless after curing, which is environmentally friendly and economical.^[^ ^22^ ^]^

Recently, Ko et al. reviewed switchable actuators and adhesives for the reconfigurable matter.^[^ ^23^ ^]^ They focused on material and device structure designs to shorten the response time, enhance the reversibility, multistimuli responsiveness, and smart adhesion for efficient shape transformation and functional actuations. Using fracture as a guide, Croll et al. discussed the fundamental mechanisms related to switchable interfaces.^[^ ^24^ ^]^ Katherine and her group reported summarized the molecular design strategies for stimuli‐responsive temporary adhesives, they concentrated on the different stimuli that may initiate debonding.^[^ ^25^ ^]^

Here, we started from the history of adhesives, describing and proposing the adhesives needed in the rapid development of the present. The following are the typical design strategies of switchable adhesive, test methods, and computer simulation methods. Then, recently switchable adhesives based on physical fields (temperature, light, magnetic field, and electrical fields) were introduced and classified. The advantages and mechanisms were discussed. We focused on the materials, switch ratio (the specific value of highest and lowest adhesion strength), switch time (time from high adhesion to low adhesion), and maximum adhesion strength (**Table** **1** **)**. Finally, future challenges and opportunities for the design and fabrication of switchable adhesives were also presented.

**Table 1:** Overview of switchable adhesives covered in this progress report

Stimuli	Materials	Maximum adhesion	Switch ratio	Switch time	Features
Light	Liquid crystal	6.1 kPa (Glass)	10.9	2 min	Light controlled tunable surface topography
	PU and graphene/shape memory polymer (GSMP)	278 kPa (Glass)	29	10 s	Light‐thermal effect controlled viscoelastic change
	Azobenzene derivatives	1.34 MPa (Aluminum)	–	180 s	Light‐thermal effect controlled phase transition
	Azopolymers	2.5 MPa (Glass)	17	5 min	Photoisomerization controlled viscoelastic change
Electric	Borate ester polymer hydrogel	12.9 kPa (Aluminum)	23	5 s	Electrolysis controlled dynamic borate ester
	Ionoelastomer	5 kPa	–	1 s	Electroadhesive
	Conductive propylene based elastomer	141 kPa (Glass)	6	30 s	Electrothermal controlled stiffness modulation
	PIL membrane with ferrocene and *β*‐cyclodextrin	80 kPa	8	10 min	Electrooxidation controlled host–guest recognition
Thermal	Recombinant protein and surfactant	600 kPa (PVC)	12	–	Thermal controlled phase transition
	Ionic crystal (IC) gel	5.82 Mpa (Glass)	–	15 min	Thermal controlled reversible crystal
	Thermal‐responsive hydrogel	142 kPa (Glass)	–	30 s	Thermal controlled LCST
	Hydrogel layer and elastomeric microcavity	94 kPa (Glass)	170	–	Thermal controlled tension and relaxation
Magnetic field	Ecoflex and iron particles	20.5 kPa (Glass)	104	0.5 s	Aphid‐inspired adhesion
	PU and NdFeB	65.9 kPa (Glass)	–	1 s	Magnetic field‐controlled negative pressure adsorption
	PDMS and Fe_3_O_4_	24.2 kPa	14	0.5 s	Magnetic field‐controlled stiffness modulation

## 2 Common Strategies and Test Simulations for Switchable Adhesion

**Figure 2:**
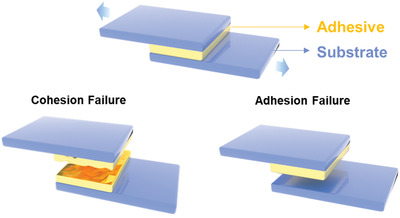
Scheme of interfacial‐adhesion and cohesion.

**Figure** **2** shows a typical switchable adhesion model. The interaction between the substrate and adhesive is referred to as the interfacial adhesion; and the other is the adhesive's strength, referred to as cohesion.^[^ ^1^, ^39^ ^]^ As with the buckets effect, the less applied force determined the system's breaking load. Thus, to make the adhesive adjustable and reversible, regulating the interfacial‐adhesion or cohesion is needed.

Interfacial‐adhesion is a result of the interaction of chemistry, topology, and mechanics.^[^ ^17^, ^40^, ^41^, ^42^, ^43^ ^]^ Therefore, it is necessary to establish interactions which play a major role in interfacial‐adhesion. Dynamic chemical bonds are well suited for controlled adhesion due to their designability and reversibility.^[^ ^44^, ^45^, ^46^, ^47^ ^]^ Under some conditions, dynamic chemical bonds (including hydrogen, ionic, and covalent bonds) can be broken and be rebuilt under conditions such as prolonged exposure to an increased temperature, electric current, and light.^[^ ^41^, ^48^ ^]^ In addition, host–guest interaction, hydrophobic interactions, and *π*–*π* stacking are also appropriate for switchable adhesion.^[^ ^49^, ^50^ ^]^ Modern individuals may also take inspiration from nature, just as ancient ones did. Octopus suckers can grasp and release things due to the pressure difference caused suction forces. By manipulating the sucker with electromagnetic fields or temperatures, the internal and surrounding pressure can be reversed for switchable.^[^ ^14^, ^51^ ^]^ Mechanical interlocking is also a force existing in the interface of adhesive and adherend. For example, gecko can scale walls mainly due to the mechanical interlocking between setae and walls.^[^ ^52^, ^53^, ^54^ ^]^ Similarly, the switchable adhesion can be achieved by controlling the interlocking.

Cohesion is the strength of the adhesive itself. In some situations, the adhesion failures are due to the break of adhesive.^[^ ^55^, ^56^ ^]^ Materials with adjustable modulus are suitable candidates for switchable adhesive. By regulating modulus, the adhesive can be turned into large adhesion or small adhesion strength. Besides, phase‐change materials, which can change states from solid to liquid, are also ideal switchable adhesives.^[^ ^57^ ^]^

**Figure 3:**
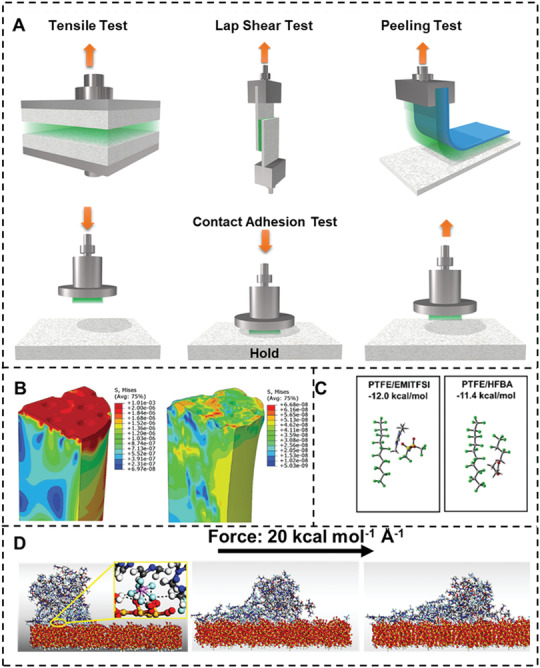
A) Common adhesive testing methods. B) Finite element analyzes simulation of stress at different temperature. Reproduced with permission.^[^ ^27^ ^]^ Copyright 2019, Wiley‐VCH. C) Density functional theory calculations evaluating binding energy between the polymer and ionic liquids. Reproduced with permission.^[^ ^63^ ^]^ Copyright 2021, American Chemical Society. D) Molecule dynamic stimulations of the adhesion process. Reproduced with permission.^[^ ^34^ ^]^ Copyright 2021, Wiley‐VCH.

The most common testing methods for adhesion include tensile test, lap sheer test, peeling test, and contact adhesion test (**Figure** **3**A).^[^ ^40^, ^58^ ^]^ In addition, surface force apparatus and atomic force microscopy can test adhesion at more minor scales.^[^ ^59^, ^60^ ^]^ As many remarkable reviews have well introduced the fundamental models and measuring instruments of adhesion,^[^ ^40^, ^41^, ^58^, ^61^, ^62^ ^]^ here we mainly focused on these testing methods corresponding switchable adhesion. The tensile test is usually used in the adsorption‐like switchable adhesion. These kinds of adhesions usually have fixed ends, and the adhesives are fixed on it to pull the adherend. In the lap shear test, the adhesion force between adhesive and adherend and the cohesion of adhesive should be considered. So, cohesion‐controlled adhesives are suitable for the lap shear test. Some adhesion depended on the adhesive and adherend, which is suitable for the peeling test. Contact adhesion tests are carried for most interfacial adhesion‐controlled switchable adhesion. Switchable adhesives usually have different states with varied adhesion strength, so that the dynamic contact test can record the constant change of the adhesion strength.

With the advent of computers, computational materials science has grown in importance in research. Typical computer simulations include numerical simulation method and theoretical simulation methods. Numerical simulation method starts from experimental data, through the establishment of mathematical model to simulate the actual process. While the theoretical simulation predicts or designs material structures and properties by the calculation of theoretical physical models.

Computational material science used in adhesion model include finite element analyze (FEA), molecule dynamics (MD), and density functionally theory (DFT).^[^ ^64^, ^65^, ^66^ ^]^ FEA is to divide the system into many small element subdomains and solve them numerically to get the quantity at each node, which is usually used to study problems related to “field,” including displacement, stress, electromagnetic, and temperature field. Tan et al. utilized FEA to investigate the deformation of the micropillars at different temperature fields (Figure 3B).^[^ ^27^ ^]^ Li and co‐workers used FEA to calculate the distribution of field intensity for magnetic stimuli.^[^ ^67^ ^]^ In addition, finite element models were also established to study the adhesion mechanisms of creatures.^[^ ^68^, ^69^ ^]^

Compared with FEA, DFT based on first‐principle is able to calculate stable structure and electronic state more accurately with a limited number of atoms. Zhang et al. studied the relationship between the chemical structure and viscosity of the supramolecular polymeric adhesive.^[^ ^63^ ^]^ Li and co‐workers calculated the combined Gibbs free energy of their adhesives and glass, which confirmed their adhesion mechanism.^[^ ^70^ ^]^ DFT was used to evaluate the binding energy between the adhesive and adherends (Figure 3C).

Molecular dynamics is a class of computer simulation methods for statistical mechanical systems, which is suitable for characterizing the interfacial structure and interaction.^[^ ^71^, ^72^ ^]^ Considering the adhesion energy equaled to the interacting energy of adhesive and adhered, the adhesion energy can be evaluated by:

(1) $$E_{\left(\mathrm{adhesion}\right)}=E_{\left(\mathrm{total}\right)}-E_{\left(\mathrm{adhesive}\right)}-E_{\left(\mathrm{adherend}\right)}$$

Here, *E*_(adhesion)_ denotes the energy of interaction between the adhesive and the substrate. *E*_(adhesive)_ and *E*_(adherend)_ represent the potential energies of adhesive and substrate, respectively. *E*_(total)_ denotes the adhesion system's total potential energy, whereas negative denotes adsorption. Further analysis of the interfacial adhesion energy can incorporate the van der Waals force and electrostatic composition, allowing researchers to understand the adhesion mechanism better. To better characterize the adhesive‐substrate interface, a continuous pulling force can be added to the adhesive in the simulation model.^[^ ^34^ ^]^ Quantifying the distance between the initial and end states and the number of hydrogen bonds created during the pulling process is possible (Figure 3D).

## 3 Light‐Controlled Adhesion

Due to their rapid cure time and excellent bonding capabilities, light‐curable adhesives have been widely employed in manual and high‐speed assembly line operations. However, as it hardens, recycling adherents face difficulties. Adhesives can be regulated by photosensitive materials.^[^ ^46^, ^73^, ^74^ ^]^ Light‐controlled adhesion is rapidly gaining attention because of its real‐time spatiotemporal control. Compared to other stimuli, light can be adjusted locally and instantly. Hohl and co‐workers concluded optically switchable adhesives in 2019,^[^ ^75^ ^]^ therefore, we mainly focused on the work published in past 3 years.

**Figure 4:**
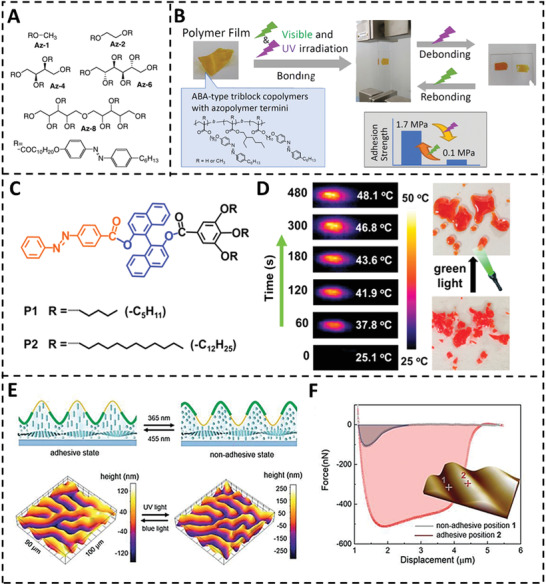
A) Chemical structures of the sugar alcohol scaffold with multi azo‐arms based light‐switchable adhesive. Reproduced with permission.^[^ ^76^ ^]^ Copyright 2012, Wiley‐VCH. B) Light‐controlled switchable adhesion based on ABA‐type triblock copolymer with azopolymer termini. Reproduced with permission.^[^ ^29^ ^]^ Copyright 2018, American Chemical Society. C) Chemical structures of the azobenzene derivatives for switchable adhesion. D) Infrared (IR) images recorded with an IR camera at different green light irradiation times and phase transition of azobenzene derivative upon green light irradiation. Reproduced with permission.^[^ ^28^ ^]^ Copyright 2019, American Chemical Society. E) Schematic illustration and 3D images of states switching via light‐triggered topographical deformation. F) Force–distance curves of the adhesive state and non‐adhesive state. Reproduced with permission.^[^ ^26^ ^]^ Copyright 2018, Wiley‐VCH.

When exposed to light, azobenzene is a widely used chemical in light‐responsive materials due to its trans–cis isomerization transition.^[^ ^77^, ^78^, ^79^, ^80^ ^]^ Akiyama et al. proposed the early light‐induced phase transition material in 2012 (**Figure** **4**A).^[^ ^76^ ^]^ The transition between the liquid and solid phases of the sugar alcohol scaffolds with multi azo‐arms was reproducible at ambient temperature with a change in the light source. Further, they exhibited the application of the multiazobenzene sugar‐alcohol derivative as switchable adhesives.^[^ ^81^ ^]^ Photoisomerization of azobenzene is generally problematic in highly ordered crystalline materials due to a lack of free volume. Except solid–liquid phase transition, photoisomerization may cause a viscoelastic property change. Ito et al. reported an ABA‐type triblock copolymer made of poly(meth)acrylates with an azobenzene moiety (A block) and a 2‐ethylhexyl (B block) side chain that has a high modulus under green light (520 nm) and a low modulus under UV light (365 nm) (Figure 4B).^[^ ^29^ ^]^ Compared to azo homopolymers, the block copolymer structure and the soft middle block improved the flexibility of the copolymers, allowing photoisomerization of the azobenzene moiety to have a more significant impact on the copolymer modulus. With glass as a substrate, this adhesive obtained shear strengths ranging from 0.1 to 1.7 MPa. In addition to photoisomerization, Wu et al. announced an azobenzene‐based light thermal‐driven solid–liquid transition in 2019 (Figure 4C).^[^ ^28^ ^]^ The azobenzene moieties agglomerate and exhibit strong absorption bands in the visible range. The produced heat causes phase changes when exposed to green light. Without the requirement for a second stimulation, the liquid‐state azo spontaneously reverts to the solid form after 2 min (Figure 4D). Feng and co‐workers reported a new approach for developing switchable adhesive utilizing adjustable surface topography in 2021.^[^ ^26^ ^]^ The technique is mostly based on light‐responsive topographical deformation. As shown in Figure 4E, the surface of the fingerprint‐configured liquid crystal network (LCN) coating is corrugated and spatially synced with the periodicity of chiral helices. The homeotropic liquid crystal (LC) orientations, with the long axes of the molecules pointing to the interface on average, are solely in elevated places, which are controlled by dichroic dye‐induced material diffusion during LC monomer polymerization. A catechol‐group‐containing adhesive polymer is spatially selectively coated on top of the LCN coating, leaving the originally upper portions with adhesive polymer and the lower parts with non‐adhesive equivalents. Trans–cis isomerization of azobenzene moieties included in the LCN causes the order parameter of the LCN to be reduced when exposed to UV light. Following this, opposing topographical reactions occur in planar and homeotropic areas: homeotropic orientation areas compress along with the LC director, whereas planar areas expand in the thickness direction, resulting in surface topography inversion. Following UV light stimulation, the sticky homeotropic portions become topographically lower than the non‐adhesive planar sections, resulting in the dynamic coating switching from adhesive to non‐adhesive (Figure 4F).

**Figure 5:**
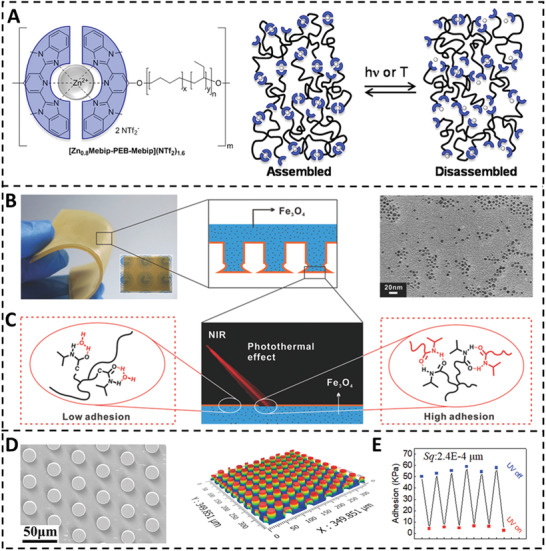
A) Schematic representation and chemical structures of the supramolecular polymers‐based switchable adhesive. Reproduced with permission.^[^ ^82^ ^]^ Copyright 2014, American Chemical Society. B) Schematic diagram and photos of the light‐controlled mushroom‐shaped polydimethylsiloxane pillar arrays decorated with poly(dopamine methacrylamide‐co‐methoxyethyl‐acrylate‐co‐N‐isopropyl acryla‐mide) p(DMA‐co‐MEA‐co‐NIPAAm) and Fe_3_O_4_ nanoparticles. C) IR‐responsive adhesion switching mechanism. Reproduced with permission.^[^ ^35^ ^]^ Copyright 2018, Wiley‐VCH. D) SEM and 3D image of resulted graphene/shape memory polymer and E) switching adhesion of GSMP on a glass substrate. Reproduced with permission.^[^ ^27^ ^]^ Copyright 2019, Wiley‐VCH.

In addition to azobenzene, Heinzmann and co‐workers developed light‐responsive supramolecular polymers that may be used as reversible adhesives (**Figure** **5**A).^[^ ^82^ ^]^ In this study, telechelic poly(ethylene‐co‐butylene) (PEB) was functionalized with either self‐complementary ureidopyrimidinone (UPy) motifs (UPy‐PEB‐UPy) or 2,6‐bis(1′‐methylbenzimidazolyl)‐pyridine (Mebip) ligands (Mebip‐PEB‐Mebip). UV light‐absorbing metal‐ligand motifs help light–heat conversion, and a UV absorber was introduced to UPy‐PEB‐UPy. They absorb UV light and heat it up, causing brief dissociation of the metal‐ligand motifs and transformation of the substance into a low‐viscosity liquid. When the light is turned off, the metallopolymers reassemble and reclaim their characteristics.

Inspired by gecko, Ma and co‐workers exhibited a photothermal controlled switchable adhesion,^[^ ^35^ ^]^ as can be seen in Figure 5B. The dynamic underwater adhesion system consists of mushroom‐shaped polydimethylsiloxane (PDMS) pillar arrays decorated with poly(dopamine methacrylamide‐co‐methoxyethyl‐acrylate‐co‐N‐isopropyl acryla‐mide) p(DMA‐co‐MEA‐co‐NIPAAm) and Fe_3_O_4_ nanoparticles. Fe_3_O_4_ nanoparticles can absorb infrared energy and heat up, making the system temperature beyond the lower critical solution temperature (LCST, about 34 °C). At this state, the PNIPAAm block exhibited a hydrophobic state, which enabled the poly(dopamine methacrylamide) better interact with the adherend, resulting a high adhesion state (Figure 5C). Tan and co‐workers demonstrated a light‐controlled adhesive made of polyurethane substrate and micropillar array of graphene/shape memory polymer composite (GSMP) via easy mould casting (Figure 5D).^[^ ^27^ ^]^ Under UV irradiation and pressure, the adhesive micropillars deformed to create conformal connections on rough surfaces. The cessation of UV irradiation vitrified the micropillars, simulating creeper secretion lignification on rough substrate. The vitrified condition of GSMP micropillars enhances homogenizes stress at the detaching contact, resulting in double the adhesion of gecko. However, laser irradiation during separation permits the simple separation of this adhesive from contacting rough surfaces. Light irradiation can therefore switch on/off adhesion (Figure 5E). This micropillar's shape memory function ensured an excellent switching ratio (29).

In conclusion, the most employed light‐controlled mechanisms are light induced isomerization transition and light‐thermal. Switchable adhesives based on these mechanisms exhibited high adhesion strength and large switch ratio. However, the lower power of light has affected the switch time. In addition, penetration depth of light also limited the application of light based adhesives.

## 4 Electric‐Controlled Adhesion

**Figure 6:**
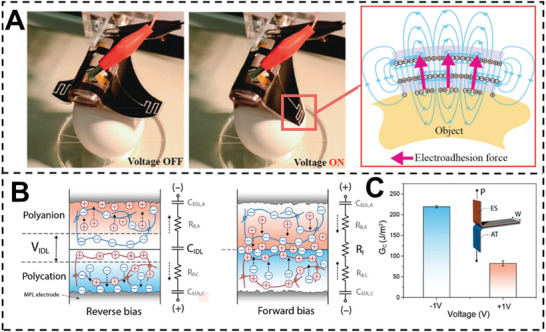
A) Picking up objects is performed using an external motorized stage where the gripper is raised and lowered by an external motorized stage to act as an end effector. Reproduced with permission.^[^ ^83^ ^]^ Copyright 2016, Wiley‐VCH. B) Schematic illustrations of a polyanion/polycation ionoelastomer junction operated under reverse bias and forward bias. Reproduced with permission.^[^ ^30^ ^]^ C) Peeling test of two heterojunctions for measuring the energy release rate. Copyright 2020, Wiley‐VCH.

In comparison to other forms of stimulation, electric‐field stimulation has many benefits, including precise controllability, rapid and reversible inducement, and easy to operate.^[^ ^84^, ^85^, ^86^, ^87^, ^88^, ^89^, ^90^ ^]^ Electroadhesion is a well‐known technique for reversible adhesion that is controlled by electric potentials and has been widely utilized in haptics and robotics. Electroadhesion usually requires a strong electric field (50–100 V m^−1^).^[^ ^91^, ^92^, ^93^ ^]^ When the electrodes are applied a high voltage, the charges on the electrodes can create fringe electric fields at the edges of the electrodes. These electric fields can cause polarization of the adherends near the electrodes, resulting in electroadhesion forces toward the electrodes. Shintake and co‐workers proposed a new dielectric elastomer gripper design based on electrostatic actuation and inherent electro‐adhesion force (**Figure** **6**A).^[^ ^83^ ^]^ Electrostatic actuation resulted in mechanical grasping with a modest force (1 mN), whereas electro‐adhesion resulted in a large holding force (3.5 N shear force per cm^2^). Switchable adhesion has a very quick process time due to the rapid switching of the electric field (about 100 ms).

However, the use of high voltages (>kV) might cause dielectric breakdown, limiting the electroadhesion's applicability. Kim et al. demonstrated a novel form of electroadhesion based on an ion elastomer junction that is controllable at low voltages (for example, one V).^[^ ^30^ ^]^ In this work, poly(ionic liquid)s (PILs) were utilized in place of dielectric elastomers. An ionic double layer may be produced at the interface between two PILs: 1‐ethyl‐3‐methyl imidazolium poly[(3‐sulfopropyl) acrylate] (ES) and poly[1‐(2‐acryloyloxyethyl)‐3‐buthylimidazolium] bis(trifluoromethane) sulfonimide (AT) (Figure 6B). The charged interfacial ionic double layer can act as an electrostatic force between two PILs when a reverse bias is applied. When the bias is reversed, mobile ions are driven into the ionic double layer and over the interface, thereby eliminating the electric field and decreasing the force of adhesion (Figure 6C). Similarly, by applying potentials to polyanion and polycation hydrogels, a concentration gradient of ions may be created, resulting in switchable adhesion.^[^ ^94^ ^]^

**Figure 7:**
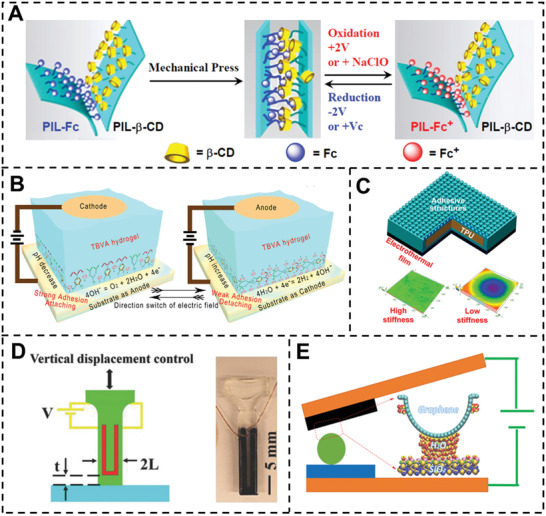
A) Schematic representation of the hook‐and‐loop strategy for adhesion based on *β*‐cyclodextrin (*β*‐CD) and ferrocene‐modified poly(ionic liquid) membrane surfaces. Reproduced with permission.^[^ ^32^ ^]^ Copyright 2014, The Royal Society of Chemistry. B) Schematic representation of mechanism for the electrically programmable adhesion of the hydrogel. Reproduced with permission.^[^ ^12^ ^]^ Copyright 2020, American Chemical Society. C) Structure and schematic of the gecko‐inspired adhesive. Reproduced with permission.^[^ ^31^ ^]^ Copyright 2020, American Chemical Society. D) Schematic of electrothermal controlled subsurface stiffness modulation and digital photo of a composite post sample. Reproduced with permission.^[^ ^95^ ^]^ Copyright 2018, Wiley‐VCH. E) Graphene based tunable adhesive force under external electrical bias. Reproduced with permission.^[^ ^96^ ^]^ Copyright 2019, American Chemical Society.

Apart from electroadhesion, electron transfer in the presence of an electric field may result in electrochemical oxidation‐reduction processes.^[^ ^97^, ^98^, ^99^, ^100^, ^101^ ^]^ Adhesives may now be regulated by an electric field, thanks to the advent of electrochemical redox‐responsive materials. Ferrocene is a well‐characterized redox‐responsive material whose redox state may be altered through redox agents or applied electrochemical potentials.^[^ ^32^ ^]^ Meanwhile, *β*‐cyclodextrin (*β*‐CD) and ferrocene derivatives can create non‐covalent host–guest interactions. Yan and co‐workers produced a pair of PIL based membranes that serve as hooks and loops, respectively, by surface grafting PIL membranes with ferrocene and *β*‐CD moieties.^[^ ^32^ ^]^ Due to the molecular recognition between the *β*‐CD and ferrocene moieties, mechanical compression can be used to attach the membranes as produced. As a result of the electron transfer, the ferrocene becomes oxidation state, which has a significantly lower affinity for *β*‐CD than the original state, thus, the applied voltage may be used to regulate the adhesion of two PIL membranes (**Figure** **7**A).

Additionally, adhesion strength can be adjusted by oxidation, such as using NaClO. Electrochemistry may also be used to regulate catechol‐based adhesives, as Bruce P. Lee demonstrated. In their study, they demonstrated the use of in situ electrical field stimulation to deactivate the adhesive properties of catechol‐containing adhesives.^[^ ^102^ ^]^ Huang and colleagues developed a borate ester polymer hydrogel whose adhesion ability can be switched fast in response to a modest electrical stimulation by changing the catechol group's exposure and shielding (3.0 and 4.5 V) (Figure 7B).^[^ ^12^ ^]^ The catechol group is exposed and shielded by reversible cleavage and reformation of the borate ester moiety caused by water electrolysis. Thus, the electric field direction may be used to regulate the hydrogel's attachment and detachment from various substrates with a reaction time as short as 1 s.

Electrothermal stimulation is another method for electric field‐induced stimulation. When a voltage is applied, the heating resistance rapidly and effectively warms up. Li and colleagues offered a switchable adhesion based on Gecko that combines the action of sticky structures with stiffness modulation (Figure 7C).^[^ ^31^ ^]^ The authors created a three‐layer adhesive that consists of a mushroom‐shaped system on top, a stiffness modulating thermoplastic polyurethane in the center, and an electrothermal film on the bottom. The top layer's ability to cling to the substrate is determined mainly by van der Waals forces generated by the surface microstructure. The top layer can alter the stiffness of the intermediate layer. While a low stiffness state allows for adequate deformation of structures to establish a conformal contact, maintaining a low stiffness state is prone to failure at the contact interface. In the state of excessive stiffness, the situation is reversed. Thus, by varying the stiffness via voltage, switchable adhesion may be achieved. Milad Tatari et al. developed a conductive propylene‐based elastomer that exhibits a substantial reduction in stiffness when triggered by an electric field (Figure 7D).^[^ ^95^ ^]^ Propylene has a transition temperature of 72.9 °C and a high Young's modulus (175 MPa) at ambient temperature but a low modulus (1 MPa) when activated. Thus, the adhesive has a stiff core at room temperature, which results in a large adhesion strength; but, when voltage is added, the core stiffness decreases, resulting in small adhesion strength.

Wan et al. established a non‐stimuli‐responsive material‐based switchable adhesion (Figure 7E).^[^ ^96^ ^]^ The electrical bias of an N‐doped graphene interface may be regulated as a durable micro/nanomanipulator in this study. When an electrical bias is provided, the graphene surface collects moisture from the air, forming water bridges between the graphene and the substrate, drastically changing the adhesion force. On the graphene/water interface, the ordered ice‐like structures reinforce the water bridges, improving force switch‐ability.

Electric‐controlled adhesion provides a simple route to rapidly and reversibly control adhesion using applied electric potentials, offering promise for various applications, including haptics and robotics. However, some electric‐controlled adhesion needs high voltages, which may cause danger. In addition, the strength of electric‐controlled adhesion still needs to be improved.

## 5 Thermal‐Controlled Adhesion

**Figure 8:**
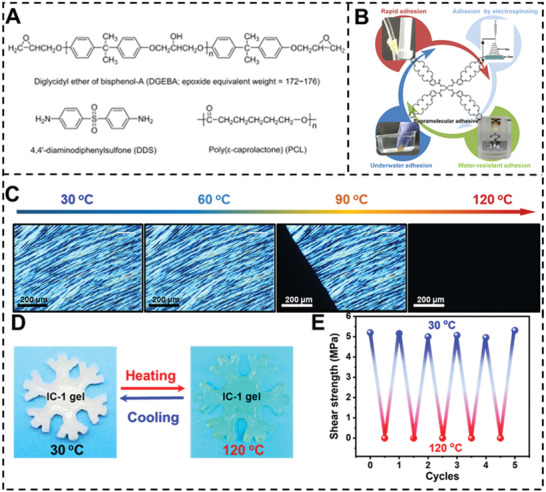
A) Chemical structures of diglycidyl ether of bisphenol‐A (DGEBA), 4,4′‐diaminodiphenylsulfone and poly(ɛ‐caprolactone) based switchable adhesive. Reproduced with permission.^[^ ^103^ ^]^ Copyright 2010, Elsevier. B) Low‐molecular‐weight supramolecular adhesives. Reproduced with permission.^[^ ^70^ ^]^ Copyright 2020, American Chemical Society. C) Polarized optical microscopy images of ionic gel at different temperatures. D) Ionic gel photographs taken at different temperatures. E) Cyclic adhesion tests of ionic gel on glass substrate. Reproduced with permission.^[^ ^34^ ^]^ Copyright 2019, Wiley‐VCH.

Switchable adhesives can also be achieved by the thermodynamic state changes (for example, from fluid to solid).^[^ ^57^, ^104^ ^]^ Phase change materials are a popular choice in the community of switchable adhesive materials due to their thermal transition characteristics.^[^ ^105^ ^]^ Mather and colleagues describe a stiff and reversible adhesive composed of a miscible mixture of bisphenol‐A/diaminodiphenylsulfone (DGEBA/DDS) epoxy and poly(3‐caprolactone) (PCL) (**Figure** **8**A).^[^ ^103^ ^]^ Fully cured material has a “bricks‐and‐mortar” structure in which densely linked epoxy spheres (“bricks”) are interpenetrated by a continuous phase PCL matrix (“mortar”). The strong bonding strength results from both efficient wettability and the subsequent production of crystalline PCL upon cooling. The PCL layer may be remelted by heating to a temperature greater than the melt point to allow debonding, demonstrating the continued availability of PCL melt adhesives.

Thermal‐response supramolecular force can also cause switchable adhesion.^[^ ^106^, ^107^, ^108^ ^]^ Li et al. created a novel low‐molecular‐weight supramolecular adhesive P1 (Figure 8B). P1 was produced by esterifying non‐viscous building blocks dibenzo‐24‐crown‐8 (DB24C8) with four‐armed pentaerythritol. P1, with a melting point of 52 °C, is a very viscous liquid that rapidly solidifies upon cooling. It adhered well to hydrophilic glass and hydrophobic polytetrafluoroethylene. The dynamic reversible supramolecular interaction prevented fatigue and decreased of P1 adhesion effect on five surfaces. The researchers created supramolecular polymers where the hydrogen bonding between the monomer PC and water molecules may induce adhesion at extremely low temperatures (−196 °C). Large water clusters are prevented by hydrogen bonding. These persistent and robust sticking properties are due to the 3D hydrogen‐bonding network formed by the water adhesive. P1 had the best adhesion in liquid nitrogen, with an average bonding strength of 1.17 MPa. High temperatures promote the development of P1‐water complexes and weaken the bonding between P1 and water molecules. Thermodynamic characteristics of P1 hydrogen bonding promote reversible adhesion. These characteristics provide P1 with strong and reversible adhesion.

More recently, Yan et al. demonstrated another phase exchange reversible adhesive by synthesizing ionic crystal (IC) gel using a photocrosslinking precursor solution consisting of *N*,*N*‐dimethylacrylamide and melted IC (Figure 8C).^[^ ^34^ ^]^ When heated to temperatures over the melting point of IC, the soft gel can be easily detached. After cooling the system, the IC gel becomes stiff, forming a thin layer of crystal IC with high cohesive strength (Figure 8D). According to their experiments, the effective adhesion strength is 5.82 MPa when the ratio of IC‐1 (*T*_m_ = 92.2 °C) to polymer is 7:3. Due to its high stickiness, IC‐1 gel (adhesion area: 2.5 × 5 cm^2^) can lift a pail weight of 51 kg. Phase change in low melting point may be utilized to alter adhesion. For example, IC‐2 gel (*T*_m_ = 65.8 °C) adhering to glass exhibited a strength of 2.73 MPa (Figure 8E).

Thermresponsive polymers have thermally switchable adhesion.^[^ ^111^, ^112^, ^113^, ^114^ ^]^ A glass or crystalline polymer has a smooth (ideally capillary wave) and hard surface. The polymer is not a strong adhesive in vitreous and crystalline states because it has limited interaction with the substrate. When heated over the glass transition temperature or melting point of the polymer, it becomes a viscous liquid with good surface contact. High viscosity polymer removal from sticky surfaces leads to significant energy waste. When the solution is cooled, the thermal expansion of the polymer and the substrate matches, resulting in strong adhesion at the interface.

**Figure 9:**
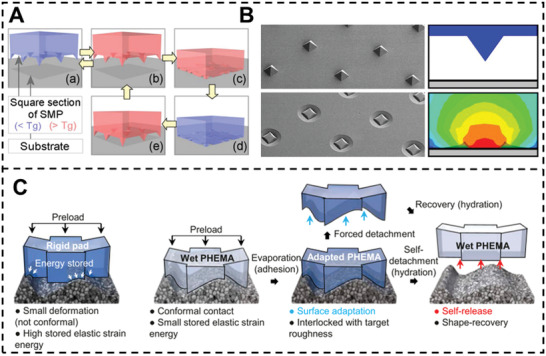
A) Schematic illustration of the bonding/debonding between shape memory polymer surface and a substrate. B) SEM and FEM images of SMP surface. Reproduced with permission.^[^ ^109^ ^]^ Copyright 2013, American Chemical Society. C) Proposed epiphragm‐like adhesion mechanism with a poly(2‐hydroxyethylmethacrylate) gel, where shape conformability in the wet state followed by interlocking upon drying facilitates adhesion. Reproduced with permission.^[^ ^110^ ^]^ Copyright 2019, National Academy of Sciences.

Shape memory polymers (SMPs) are thermosensitive adhesives designed to significantly change their modulus at the glass transition temperature.^[^ ^115^ ^]^ Kim and colleagues used thermally switchable adhesive with small pyramid‐shaped columns (**Figure** **9**A). Since these columns significantly limited contact between the polymer and the glass substrate, adhesion is relatively poor. When heated, the polymer deforms and comes into close contact with the substrate, which is then trapped within the polymer upon cooling. The sample can be heated to restore the pyramids to their low adhesion state. It possesses a rapid switching rate and switchable adherence to glass substrates (strong/weak adhesion ratio > 1 × 10^4^). While the technique works well on a flat surface, it suffers on a rougher one (Figure 9B). With the use of poly(2‐hydroxyethylmethacrylate) (PHEMA) hydrogel's shape adaptability and memory capabilities, Yang and colleagues created an intrinsically reversible, snail epiphragm‐inspired adhesive with a quasi‐strong sticky (up to 892 N·cm^2^) (Figure 9C).^[^ ^110^ ^]^ It works well on both smooth and uneven surfaces. Its adhesion strength is structure independent, and its near‐surface elastic modulus ranges from 180 kPa hydrated to 2.3 GPa dry, similar to snail mucus. The PHEMA gel conforms to the surface via low energy deformation and locks as the elastic modulus climbs from hundreds of kPa to 2.3 GPa. Since the shape‐adapting adhesion is not dependent on the reversible adhesive's geometry, it may be used on a variety of substrates.

Thermal‐controlled adhesion is one of the most commonly used switchable adhesion strategies. Many other stimuli can also turn into thermals to achieve switchable adhesion. These adhesives have high adhesion strength and switch ratio. However, heating is relatively slow process, resulting in a long switch time.

## 6 Magnetic Field‐Controlled Adhesion

**Figure 10:**
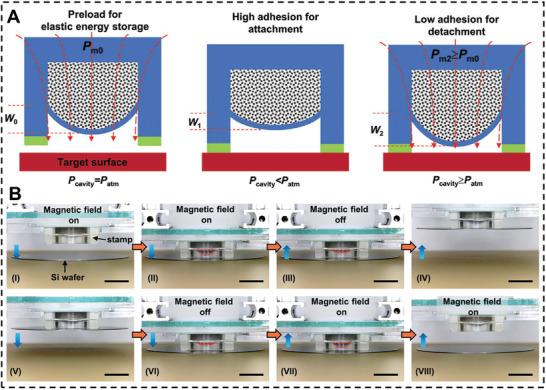
A) The elastic energy storage enabled magnetically‐actuated, octopus‐inspired smart adhesive. B) Demonstrations of the octopus‐inspired smart adhesive for manipulating common objects with various surfaces. Reproduced with permission.^[^ ^116^ ^]^ Copyright 2021, Wiley‐VCH.

Fast reaction, noncontact, and stable magnetic field‐induced adhesions are based on bio‐inspired actuators with surface structure control.^[^ ^67^, ^117^, ^118^, ^119^, ^120^, ^121^ ^]^ Wang et al. created a magnetically operated adhesion device with rapid responsiveness and low energy consumption (**Figure** **10**A).^[^ ^116^ ^]^ An elastic membrane separates two chambers in the adhesive. The top cavity contains magnetic particles while the lower is empty. Initially, cavity pressure (*P*_cavity_) equals atmospheric pressure (*P*_atm_). The deformation of the elastic membrane caused a preloading magnetic pressure. The magnetic field is withdrawn and the stored elastic energy pulls the membrane higher, reducing *P*_cavity_. *P*_cavity_ is less than *P*_atm_, resulting in a strong adhesion condition. Applying a magnetic field again can raise the *P*_cavity_, creating a poor adhesion condition. The adhesion system obtained an energy efficiency by using elastic energy storage (Figure 10B). Similarly, Hu et al. demonstrated a magnetic suction cup based on Ecoflex with a 0.5 s switch time. The cavity's internal and exterior pressure differential generates adhesion.^[^ ^119^ ^]^

**Figure 11:**
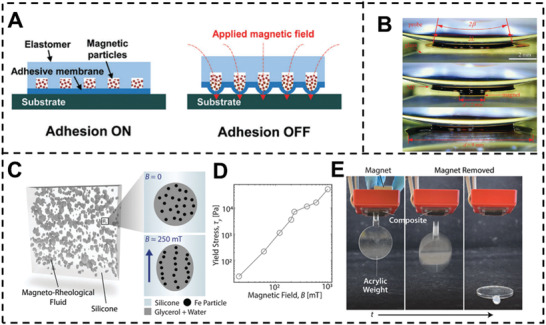
A) Adhesion ON state with a flat adhesive membrane and OFF state with a deformed adhesive membrane under the applied magnetic field. Reproduced with permission.^[^ ^36^ ^]^ Copyright 2019, The Royal Society of Chemistry. B) Side views of the MF meniscus between probe and glass substrate with and without the magnetic stimuli. Reproduced with permission.^[^ ^37^ ^]^ Copyright 2019, The Royal Society of Chemistry. C) X‐ray tomogram of the magneto‐rheological fluid–silicone composite. D) Measured yield stress of the magneto‐rheological fluid–silicone composite with applied magnetic field. E) Demonstration of magnetically‐switchable adhesion. Reproduced with permission.^[^ ^38^ ^]^ Copyright 2019, The Royal Society of Chemistry.

Linghu et al. proposed an approach based on elastomer and magnetic particles where adhesion is controlled by the surface topography (**Figure** **11**A).^[^ ^36^ ^]^ A thin surface membrane encloses a hollow filled with magnetic particles. Sticking to a substrate required a flat surface membrane in the adhesion ON state. When the magnetic particles were subjected to a magnetic field, they were magnetized, causing the thin surface membrane to swell around the interface. With continuous application of the magnetic field, the surface membrane began to peel at the outside perimeter. It propagated to the center, lowering the contact area and interfacial adhesion, a phenomenon referred to as the adhesion OFF state. This magnetic force‐dependent adhesion device exhibited a high switch ratio (104), a fast tuning time (0.5 s), and a high reversibility (50 cycles). Drotlef and colleagues constructed a magnetic micropillar array using PDMS precursors containing NdFeB microparticles. With the applied magnetic field, the micropillars may be bent and spin around their own axis. The magnetic field may convert the surface characteristics between sticky and non‐adhesive states based on this topographical alteration and bioinspired adhesive pillar design.

Apart from magnetic particles and elastomer‐based composite materials, magnetic fluid (MF) was also utilized to provide programmable multiscale topography. Li et al. created an innovative adhesion system using Fe_3_O_4_MFs based MFs.^[^ ^37^ ^]^ The capillary force between two solid surfaces may be raised or lowered by adjusting the applied magnetic field strengths distribution, resulting in a switchable adhesive characteristic (Figure 11B).

Testa and colleagues used a magneto‐rheological fluid (MRF) comprised of carbonyl iron particles (80% by weight) in a glycerol solution (50 wt%) to produce switchable adhesion (Figure 11C).^[^ ^38^ ^]^ The MRF was first distributed in silicone polymeric precursor solution to form MRF droplets. After the silicone crosslinking, the MRF droplets became trapped in elastic matrixes, allowing the MRF Young's modulus to be adjusted between 4 Pa and 40 kPa (Figure 11D). The MRF increased dissipation in bulk, increasing the composite's toughness and impeding the development of interfacial fractures. Thus, the magnetic field enables the high adhesion condition to be attained (Figure 11E).

Magnetic field‐controlled responsive materials are characterized by a fast response, non‐contact, and stable properties thus have significant advantages in switchable adhesion. Switchable adhesion can be easily achieved by controlling the surface topography or shapes by the magnetic field. However, there are few options for realizing magnetic field‐controlled adhesion, which still need to be explored and expanded.

## 7 Other‐Controlled Switchable Adhesion

### 7.1 pH‐Controlled Adhesion

**Figure 12:**
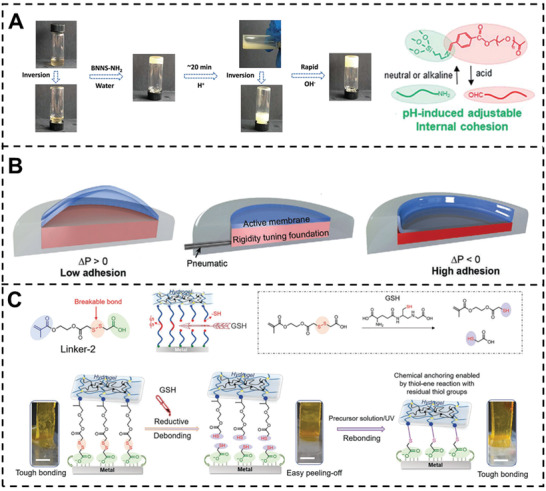
A) The dynamic bonding and debonding processes of pH‐induced hydrogel. Reproduced with permission.^[^ ^125^ ^]^ Copyright 2020, The Royal Society of Chemistry. B) Rapidly switchable adhesives through active membrane‐foundation adhesives (AMFAs). Schematic of an AMFA in the low adhesion and high adhesion states. Reproduced with permission.^[^ ^126^ ^]^ Copyright 2021, Wiley‐VCH. C) Tough bonding, on‐demand debonding, and facile rebonding between hydrogels and metal surfaces via the dynamic linker. Reproduced with permission.^[^ ^127^ ^]^ Copyright 2019, Wiley‐VCH.

Weak alkaline groups, which can be protonated or deprotonated, are usually utilized for preparing pH‐responsive materials.^[^ ^46^, ^122^, ^123^, ^124^ ^]^ Driven by the Schiff base chemistry and catechol groups, Jin et al. developed a pH‐controlled adhesive.^[^ ^125^ ^]^ As shown in **Figure** **12**A, the dynamic network is composed of amino‐decorated boron nitride nanosheets and aldehyde group‐terminated PEG side chains. Under alkaline condition, amino‐decorated boron nitride nanosheets can act as cross‐linkers to maintain the network. Once adjusting the pH from 9 to 3, the debonding of imine linkages liquefaction caused the liquefaction of the adhesive, making the decrease of cohesion. Thus, the adhesion strength of this adhesive can be adjusted from ≈1.44 to 0.30 MPa in 20 min. Arias et al. synthesized an artificial mussel‐glue protein by tyrosinase‐activated polymerization. The *β*‐sheets are suppressed under the pH 5.5 and regained as the pH varies from 5.5 to 6.8, resulting in the change of the cohesion. In situ switch experiments showed that the adhesion energy varied from 0.60 to 1.80 MJ m^−2^.

### 7.2 Mechanical Process‐Controlled Switchable Adhesion

Mechanical processes usually involve wrinkling, swelling, stretching, etc. Most of the destructions of adhesives are mechanical processes but cannot be reversible. However, skillful use of some mechanical processes can also make the adhesion switchable.^[^ ^128^, ^129^ ^]^ Surface wrinkles have been widely utilized to enhance the adhesion. Li et al. proposed an approach for switchable adhesion by regulating wrinkles.^[^ ^130^ ^]^ Two elastic hydrogel adherends were bonded via a wrinkled hydrogel adhesive. The wrinkle of the hydrogel adhesive can be regulated by pre‐stretch. By regulating the wrinkle, the adhesion can be improved due to restrained crack propagation or decreased by the suppression of wrinkles. Swift and co‐workers reported a pneumatically controlled switchable adhesion with a high switch ratio (about 1300) and short switch time (0.1 s).^[^ ^126^ ^]^ The switchable adhesion system consisted of an active PDMS membrane and rigidity tunable foam foundation. Upon a positive pressure, the active membrane inflated, and the adhesion was decreased. While, the increase of adhesion can be achieved by entering a negative pressure (Figure 12B). Using PDMS and glass beads, Ohzono and co‐workers exhibited a strain based switchable adhesion.^[^ ^131^ ^]^ The glass beads were placed on the uncured PDMS sol and the beads submerged and reached the bottom of the PDMS under the effect of gravity. After curing, the bead‐embedded PDMS elastomer was obtained. The elastomer was flat at the original state, while once the elastomer was stretched, bumps formed by the beads can reduce the contact area between the elastomer and the adherend, causing a decrease in adhesion.

### 7.3 Redox‐Controlled Switchable Adhesion

To achieve specific molecule‐controlled switchable adhesion, Li and co‐workers provided a universal approach for hydrogels bonding with metals.^[^ ^112^ ^]^ As shown in Figure 12C, a linker molecule with a carboxylic acid group and disulfide bonds were modified on the interface between metal and hydrogel and the linker molecule can be reduced by reducing agent, such as glutathione (GSH). As the adding of GSH, the dynamics disulfide bonds broke, causing the debonding of metal and hydrogel. After the reductive debonding, the resulted metal surface with free thiol groups can be easily rebonded with a new hydrogel again. Thus, on demand bonding and debonding can be achieved.

## 8 Summary and Outlook

As the development of science and technology, the applications of switchable adhesives have been greatly expanded. Typically, flexible electronics and soft robotics, have exhibited significant requirements and opportunities for next generation of switchable adhesives. Here, we started from the history of adhesives, describing and proposing the adhesives needed in the rapid development of the present. Then, we discussed the typical design strategies of switchable adhesive and the test and computer simulation methods of switchable adhesion. Finally, we summarized the physical fields (temperature, light, and electric and magnetic field) controlled switchable adhesion. The methods of these works are fascinating and impressive. However, challenges and opportunities still exist in switchable adhesion fields.

One challenge is to achieve switchable adhesion with fast switching speed, high switch ratio, and large adhesion strength simultaneously. For example, switchable adhesives controlled by electromagnetic fields usually have a fast switching speed and high switch ratio, while the adhesion strength is relatively weak. On the other hand, thermo and light‐controlled adhesives usually have a relatively higher adhesion strength, however, with a longer switching time. Obviously, single physical field controlled switchable adhesion cannot achieve fast switching speed, high switch ratio, and large adhesion strength simultaneously. Therefore, multiple stimuli should be integrated to simultaneously achieve this goal.

Another challenge is to realize fine control of adhesion strength. For example, most of switchable adhesions have two states, on‐state for high adhesion strength and off‐state for weak adhesion strength. At the same time, some cases, such as gripers and robots, need adjustable forces to grip objects. Excessive adhesion force may cause damage to adherents. In order to achieve this goal, more precise physical fields’ regulation should be carried out. Besides, the accurate control of adhesion force can also be reflected in the choice of adherents. Adhesives that can bond specific materials under physical fields will also be a challenge.

The third challenge is to carry out the nondestructive testing of adhesion strength. Most adhesion strength tests are based on failure mechanisms, which utilize destruction to confirm adhesion strength. However, once the adhesive has been bonded, detection of adhesion strength by destruction is not an appropriate solution. In this case, the relationship between adhesion strength and sensing signals can be modeled carefully. Thus, the adhesion strength can be sensed.

Opportunities and challenges coexist. First, new stimulus can be introduced contiouslly. For example, as a mechanical wave, ultrasound has been used for nondestructive testing of solid materials. Compared with electromagnetic wave, ultrasound‐controlled adhesive may achieve switchable inside the solids. As a commodity, there is a considerable amount of adhesive used world widely. Most adhesives, however, are nondegradable and non‐reusable. Switchable and reversible adhesion provided a path to make adhesives reusable, which is beneficial for the environment. More than that, environment‐friendly materials and degradable materials should be used in adhesives. Finally, we hope that sustainable development of switchable adhesion systems can promote more broad application prospects.

## Conflict of Interest

The authors declare no conflict of interest.
